# *Acinetobacter* phage genome is similar to Sphinx 2.36, the circular DNA copurified with TSE infected particles

**DOI:** 10.1038/srep02240

**Published:** 2013-07-19

**Authors:** Toshisangba Longkumer, Swetha Kamireddy, Venkateswar Reddy Muthyala, Shaikh Akbarpasha, Gopi Krishna Pitchika, Gopinath Kodetham, Murali Ayaluru, Dayananda Siddavattam

**Affiliations:** 1 Department of Animal Sciences, School of Life Sciences, University of Hyderabad, Hyderabad – 500 046, India; 2 Dept. of Plant Sciences, School of Life Sciences, University of Hyderabad, Hyderabad – 500 046, India; 3 Bioinformatics Centre, Pondicherry University, Puducherry - 605 014, India

## Abstract

While analyzing plasmids of *Acinetobacter* sp. DS002 we have detected a circular DNA molecule pTS236, which upon further investigation is identified as the genome of a phage. The phage genome has shown sequence similarity to the recently discovered Sphinx 2.36 DNA sequence co-purified with the Transmissible Spongiform Encephalopathy (TSE) particles isolated from infected brain samples collected from diverse geographical regions. As in Sphinx 2.36, the phage genome also codes for three proteins. One of them codes for RepA and is shown to be involved in replication of pTS236 through rolling circle (RC) mode. The other two translationally coupled ORFs, *orf106* and *orf96*, code for coat proteins of the phage. Although an *orf96* homologue was not previously reported in Sphinx 2.36, a closer examination of DNA sequence of Sphinx 2.36 revealed its presence downstream of *orf106* homologue. TEM images and infection assays revealed existence of phage AbDs1 in *Acinetobacter* sp. DS002.

A*cinetobacter* sp. are of great interest owing to the diverse habitats they colonize, the involvement of certain strains in epidemic outbreaks in hospitals and their various metabolic capabilities like aromatic catabolism, degradation of hydrocarbons in oil spills etc^1^,^2^,^3^. *Acinetobacter* *baumannii* is known to be an opportunistic pathogen and is implicated in nosocomial infections, bacteremia, secondary meningitis, urinary tract infections and ventilator-associated pneumonia etc^4^. Since 1970's *A.* *baumannii* has been known to have a clear role in multi-drug resistance. The ever-increasing resistance of *A.* *baumannii* against many clinically important antibiotics has been attributed to its proclivity in acquiring the drug resistance genes from among the members of the microbial community^5^,^6^. Dissemination of the resistance genes occurs mainly through horizontal gene transfer (HGT)^7^,^8^,^9^,^10^. Plasmids play a predominant role in HGT and the existence of plasmids carrying genes of high ecological and physiological relevance has been reported for *A. baumannii*^5^,^11^,^12^,^13^. In view of its clinical significance, the complete genome sequence has been determined for a number of *A. baumannii* strains isolated from different geographical regions and in most of them the existence of multiple plasmids has been reported^14^,^15^,^16^,^17^.

Transmissible spongiform encephalopathies (TSEs) such as Creutzfeldt–Jakob Disease (CJD) and kuru in humans, scrapie in sheep, and BSE in cows are caused by a group of related, but incompletely characterized infectious agents. Prions are responsible for the TSE in a variety of mammals^18^,^19^. In all healthy animals, prions (PrP^C^) are found in the membranes of all the cells and distributed throughout the body. However, Prions (PrP^Sc^) found in TSE-affected animals have an amyloid fold and are resistant to proteases^18^. Although the precise reasons for conversion of PrP^C^ to PrP^Sc^ are unknown, the PrP^Sc^ has been shown to induce conversion of PrP^C^ into PrP^Sc^. The prion-only theory suggests that no external agent is involved in conversion of PrP^C^ to PrP^Sc^^18^. In contrast to this notion, however, a number of studies have implicated an environmental origin of TSE agents due to geographic prevalence and occurrence^20^,^21^,^22^. Studies have also indicated their transmission through the gastrointestinal tract and blood^23^,^24^,^25^. A recent study has identified two circular DNA molecules in the TSE particles purified from infected samples collected from diverse geographical regions^26^. These two circular DNA molecules were designated as SPHINX sequences using an acronym given for Slow Progressive Hidden INfections of variable (X) latency as they were enriched in infectious preparations. Of these two Sphinx sequences, Sphinx 1.76 has shown 70% sequence similarity to a plasmid, p1ABTCDC0715 isolated from *A.* *baumannii* TCDC-AB0715^27^ and a comparable similarity to a plasmid, p2ABAYE found in *A. baumannii* AYE^13^. However, no good homologues were found for Sphinx 2.36, the second Sphinx sequence isolated from BSE-infected samples. Sequence similarity was only seen in the DNA region that codes for a replication protein RepA^26^. In the present study we report the existence of a circular double-stranded DNA in *Acinetobacter* sp. DS002, with high sequence similarity to Sphinx 2.36. Sequence similarity of 67% between these two DNA molecules was found both in coding and non-coding sequences. There was absolute sequence identity in the region containing replicative elements, such as the double-stranded origin of replication (DSO) and the RepA-coding sequence. The experimental evidence presented in this study suggests that the circular DNA is the genome of a phage capable of replicating via the rolling-circle mode.

## Results

**Figure 1:**
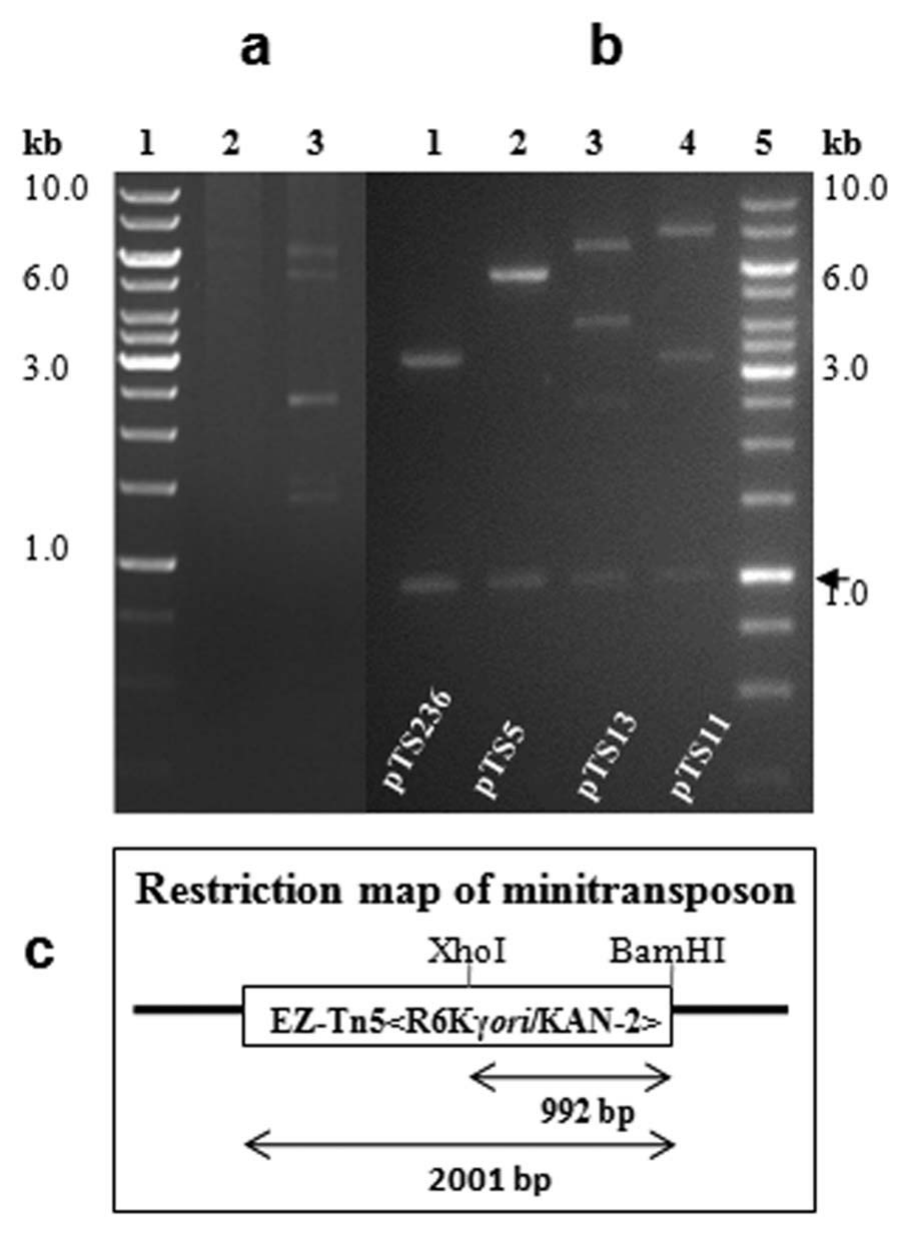
In panel (a) an agarose gel shows the existence of multiple plasmids in *Acinetobacter* sp. DS002. Lane 1 and 2 represent a kilobase DNA ladder as standard and plasmid preparations from the plasmid-free *E. coli* strain used as negative control, respectively. The plasmids found in *Acinetobacter* sp. DS002 are shown in lane 3. Panel (b) shows the restriction profile of rescue-cloned plasmids of *Acinetobacter* sp. DS002 isolated from *E. coli* pir116 cells. The plasmids were independently digested with XhoI and BamHI and analyzed on 0.8% (w/v) agarose gels. The total size of the rescue-cloned plasmids was calculated based on the electrophoretic mobility of the DNA fragments generated upon restriction digestion. A portion of the minitransposon released as a XhoI and BamHI fragment is shown with an arrow. While calculating the total size of rescue-cloned plasmids a size of 2 kb based on the size of the minitransposon was deduced. Panel (c) shows the restriction map of the minitransposon-EZ-Tn5<R6Kγ*ori*/KAN-2> inserted into rescue-cloned plasmids. The open box represents the minitransposon and the lines flanking the open box indicate the rescue-cloned plasmid. The size of the minitransposon and the 1.0 kb portion released upon digesting with BamHI and XhoI is indicated with inverted arrows.

A number of *Acinetobacter* sp. have been shown to have multiple plasmids with different sizes^17^. Some of them, especially strains of *A. baylyi*, have the ability to take up linear and circular DNA molecules under natural conditions^28^. Such an unique ability is attributed to the acquisition of multiple plasmids and resistance genes for multiple drugs from microbial communities^6^. We have been studying regulation and horizontal mobility of organophosphate-degrading (*opd*) genes among soil bacteria^29^,^30^. *Acinetobacter* sp. DS002 was isolated from OP pesticide-polluted agricultural soils and the strain name was given based on 16 S rRNA gene sequence identity (100%) with the type strain *Acinetobacter baumannii* MDR-TJ. The culture deposited in Microbial Type Culture Collection Center (MTCC), IMTECH, Chandigarh, India is available as *Acinetobacter* sp. DS002 MTCC11451. *Acinetobacter* sp. DS002 was originally analyzed to detect extra chromosomal genetic material, to establish a link, if any, between the plasmids and OP pesticide degradation. Four indigenous plasmid molecules were detected (Fig. 1a) in this soil isolate and were designated as pTS13 (13 kb), pTS11 (11 kb), pTS5 (5 kb) and pTS236 (2.2 kb). Initial hybridization studies using a well-conserved organophosphate-degrading (*opd*) gene, gave no signal with any of the detected plasmids or with the chromosomal DNA suggesting that the OP degradation mechanism of *Acinetobacter* sp. DS002 might be novel. In the process of identifying *opd* plasmids through complementation, we rescue-cloned the plasmids of *Acinetobacter* sp. DS002 into *E. coli* *pir*116 cells after tagging with a mini-transposon (EZ-Tn5<R6Kγ*ori*/KAN-2>), which has a R6Kγ replicative origin. The unique restriction profiles and size indicated that rescue cloning of all identified circular DNA molecules from *Acinetobacter* sp. DS002 had been successful (Fig. 1, panel b). When sequenced, one of the rescue-cloned plasmids, pTS236, showed significant sequence similarity (67%) to the recently reported Sphinx sequence, Sphinx 2. 36.

### Sequence analysis of pTS236

The 2252 bp sequence of pTS236 is deposited in the EMBL GenBank and can be accessed with the number JN872565. The open reading frames (ORFs), putative promoter and inverted and imperfect repeats predicted in pTS236 are shown in Supplementary Fig. S1. Only three ORFs were identified in the sequence of pTS236. One of them showed sequence similarity to *repA*, which is generally found in plasmids that exhibit rolling-circle replication. The other two ORFs that code for proteins 106 and 96 amino acids in length had no sequence similarities to any known protein sequences available in the databases and hence were designated as *orf106* and *orf96*. These two ORFs appear to be translationally linked, as the predicted translational stop codon of *orf106* overlaps with the start codon of *orf96*. Moreover, a consensus σ^70^-dependent promoter was identified only in the upstream region of *orf106* and no predicted promoter sequences were found upstream of *orf96* (Supplementary Fig. S1). The absence of promoter elements upstream of *orf96* and the existence of translational coupling strongly suggested these two ORFs formed an operon (Supplementary Fig. S1). While gaining experimental evidence, the total RNA extracted from *Acinetobacter* sp. DS002 cells was used as template and RT-PCR was performed using *orf106*-specific forward and *orf96* reverse primers. An amplicon equivalent to the size of these two ORFs was amplified indicating that these two adjacent ORFs are organized as an operon (Supplementary Fig. S2).

A 12 bp long sequence that matched perfectly the double-stranded origin of replication (DSO) typically found in rolling-circle (RC) replicating plasmids was found in the intergenic region between *orf96* and *repA* (Supplementary Fig. S1). Indeed, the −10 hexameric sequence of a putative σ^70^-dependent promoter of the *repA* gene was located in the middle of the DSO sequence motif (Supplementary Fig. S1).

### RepA translation starts from the UUG codon

**Figure 2:**
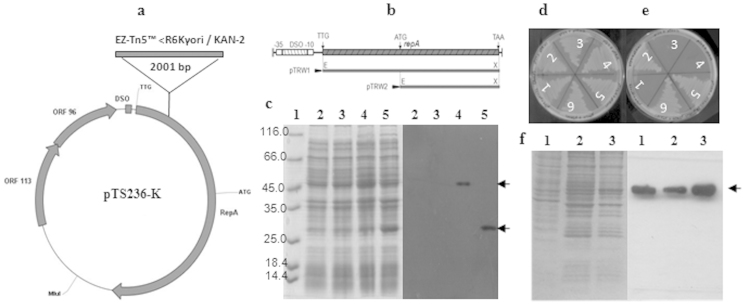
Panel (a) shows the pTS236-K map with a minitransposon insertion between the predicted start codon UUG and a single AUG codon of *repA* specifying 165-methionine residue. Arrows indicate the transcriptional orientation of *repA*, *orf106* and *orf96*. The DSO found upstream of *repA* start codon UUG is shown as a filled square. Panel (b) shows the physical map of the DSO and *repA* region of pTS236. DSO is shown as a hatched box. The extent of *repA* cloned to generate expression plasmids coding RepA (pTRW1) from predicted start codon UUG and from the AUG specifying 165-methionine (pTRW2) are indicated with solid lines. Panel (c) shows an SDS-PAGE and the corresponding western blot probed using anti-His antibody. Lane 1 represents the protein molecular mass marker. Lanes 2 and 3 show protein extracts prepared from uninduced *E.* *coli* BL21(DE3) cells having either pTRW1(lane 2) or pTRW2 (lane 3). Lanes 4 and 5 represent similar extracts prepared from induced cultures. RepA-specific signals seen in induced cultures are shown with arrows. Panel (d) and (e) represent replication of pTS236-K in permissive (*E. coli* pir116) and non-permissive (*E. coli* BL21) hosts. Panel (d) shows growth on LB with ampicillin and kanamycin plates of *E. coli* pir116 (pTS236-K) containing expression vector pET23b (sector 1), pTRW1 (sector 2), pTRW2 (sector 3), pTRM (sector 4). Sectors 5 and 6 represent growth of *E. coli* pir116 (pTRW1) having plasmids pT106M and pT96M, respectively. Growth of *E. coli* BL21 carrying the same plasmids is shown in panel (e). Panel (f) shows an SDS-PAGE and the corresponding western blots generated using RepA specific antibodies. Lane 1 represents total proteins of *Acinetobacter* sp. DS002. Lanes 2 and 3 are protein extracts prepared from *E. coli* BL21 cells expressing RepA from predicted start codon with or without C-terminal histidine tag, respectively.

The bioinformatic tool GLIMMER, which is used to predict ORFs, has indicated that initiation of RepA translation occurs at an UUG start codon, which is located 21 bp downstream of the putative promoter element of *repA*. The first AUG codon was found 513 bp downstream of the putative promoter element. The predicted amino acid sequence coded by the DNA region present between these two codons shows strong sequence similarity to the N-terminal portion of the RepA sequence coded by a well-characterized RC plasmid pPP81^31^, strengthening the supposition of a possible initiation of RepA synthesis from the UUG codon. As this is an unusual observation, we next determined whether RepA is indeed translated from the UUG codon. We initially collected all rescue-cloned pTS236 plasmids with a single mini-transposon insertion and established their restriction profile to locate precisely the point of mini-transposon insertion. In one of the rescue-cloned pTS236 derivatives, the mini-transposon was inserted between predicted start codon UUG of RepA and the conventional start codon AUG (Fig. 2a). This pTS236 variant, pTS236-K failed to replicate in *pir* negative *E. coli* cells (Fig. 2e). This derivative was therefore used to develop a complementation assay to assess the functional status of RepA expressed from these two putative translational start codons. Two expression plasmids were constructed by complementing with an expression plasmid encoding RepA either from the predicted start codon UUG (pTRW1) or from the conventional start codon AUG (pTRW2). Initially these expression plasmids were transformed into *E. coli* BL21(DE3) cells and were used as host to co-transform with plasmid pTS236-K. When selected on kanamycin plates, which indicates the ability of pTS236-K to replicate, growth occurred only with *E. coli* BL21 (pTRW1) cells encoding RepA initiating from codon UUG (Fig. 2d, e). No growth was found in *E. coli* cells with RepA encoded from AUG, suggesting that the N-terminal portion of RepA specified by the sequence found upstream of the AUG is essential for the RepA function. Supporting this result, a signal similar to the size of RepA coded from codon UUG was obtained when total proteins of *Acinetobacter* sp. DS002 were probed with RepA specific antibodies (Fig. 2f).

### Tyrosine 265 is essential for RepA function

Plasmids that replicate using the rolling-circle mechanism have a functionally conserved tyrosyl residue near the C-terminus (Supplementary Fig. S3). This invariant tyrosyl residue has been implicated in initiation of the replication process by generating a nick at DSO^32^. The RepA of pTS236, when aligned with the RepA of pPP81^31^, a well characterized RC plasmid, as well as with RepA proteins from other RC plasmids isolated from *Staphylococcus* *aureus*^33^,^34^ and *Lactobacillus* *hilgardii*^35^, have a high similarity throughout the protein, particularly in the segment with the conserved tyrosyl residue (Supplementary Fig. S3). In order to assess if the conserved tyrosine of RepA plays a role in replication of pTS236, we performed a complementation assay by transforming plasmid pTS236-K into *E. coli* BL21 cells having expression plasmids coding for either wild type (pTRW1) or mutant (pTRM) RepA proteins (Fig 2 d, e). Only BL21 with the plasmid pTRW1 exhibited kanamycin resistance suggesting that replication of plasmid pTS236-K was dependent on the invariant tyrosine residue. A similar complementation assay was performed to determine whether the ORFs *orf106* and *orf96* have any role in the replication process. Variants of pTS236-K with a translational termination codon incorporated immediately downstream of the predicted start codon of either *orf106* or of *orf96* successfully replicated in *E. coli* BL21cells (pTRW1) (Fig. 2e). This indicates that neither ORF is required for replication of pTS236-K.

### RepA DSO interactions

**Figure 3:**
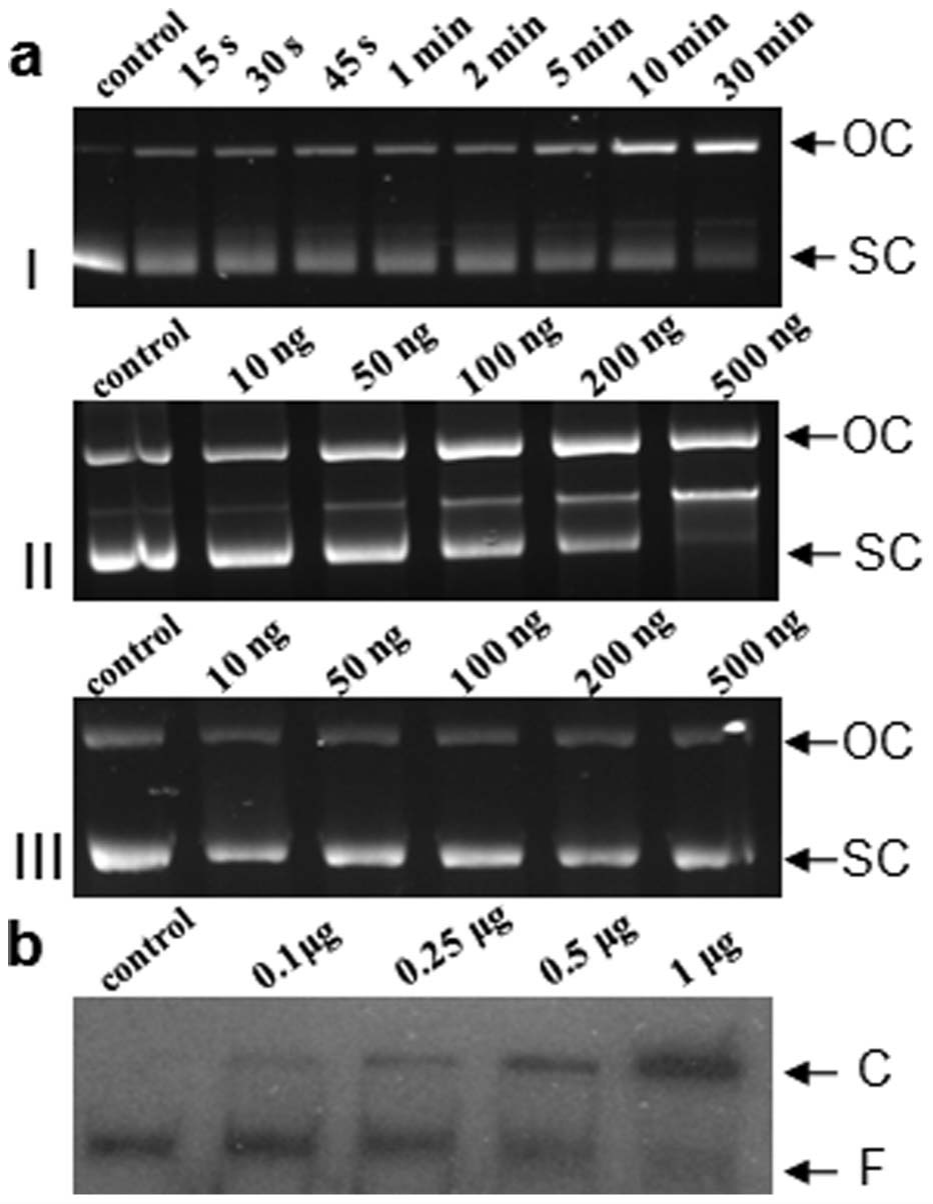
RepA-mediated generation of open-circular (OC) form of plasmid pTS236-K from a super-coiled (SC) form. Panel (a) indicates SC to OC conversion of pTS236-K against time (I) or with increasing concentration of RepA (II). The pTS236-K incubated with increasing concentrations of RepA(Y265F) is shown in panel (a) III. Electrophoretic Mobility Shift Assay (EMSA) for DSO and RepA is shown in panel (b). A shift in DSO mobility was observed due to the formation of DSO-RepA complex when labelled DSO was incubated with increased concentrations of RepA. Control represents ^32^P-labelled DSO without RepA. The DSO mobility shift is shown with an arrow (C) while F represents the free probe.

**Figure 4:**
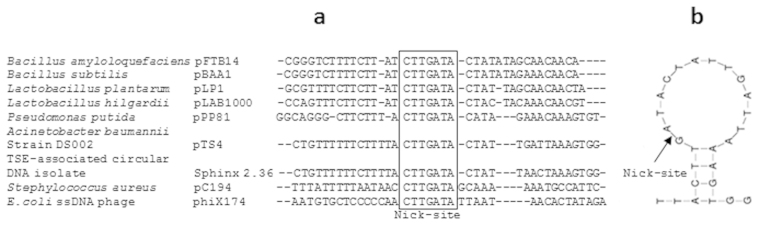
Alignment of Double-Stranded Origin of replication (DSO) of plasmid pTS236 with the known rolling circle replicating plasmids (Panel a). Panel (b) shows the secondary structure of DSO. The proposed site of the nick is shown with an arrow.

**Figure 5:**
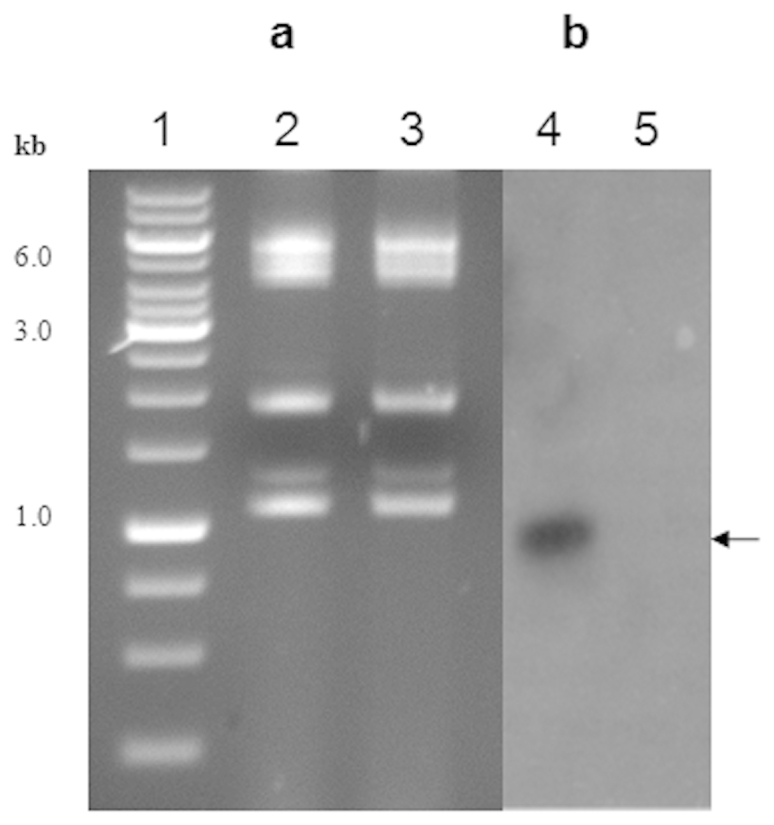
Detection of the single-stranded intermediate of pTS236: Lane 1 represents 1 kb DNA ladder, lanes 2 and 3 represent total plasmid preparation treated with (2) and without (3) S1 nuclease. Lanes 4 and 5 represent the corresponding autoradiogram developed using labelled pTS236 as probe. Single-stranded pTS236 is shown with an arrow mark.

In order to assess any interaction of RepA with the predicted DSO of pTS236, recombinant RepA_6His_ was purified and used to perform two independent *in vitro* studies. Initially recombinant RepA_6His_ was incubated with the supercoiled form of pTS236-K and the ability of it to form the OC species of the plasmid was analyzed. Generation of the OC form of pTS236-K increased both in a time- and concentration-dependent manner upon incubation with RepA and no conversion was seen in control samples prepared if RepA was omitted (Fig. 3a). Though the data clearly suggests a possible RepA-dependent generation of the OC species from the supercoiled plasmid, it does not indicate whether the generated nick was in the DSO region. Initially, about 100 bp of sequence flanking the predicted DSO was amplified using primers DS00101and DS00102 and the ^32^P end-labeled amplicon was used to perform electrophoretic mobility shift assay (EMSA) by incubating with pure recombinant RepA_6His_. An apparent shift was observed in the mobility of the amplicon due to mobility retardation of the DSO-RepA complex (Fig. 3b). The complex was not dissociated in the presence of BSA or herring sperm DNA. Suggesting that the interactions between DSO and RepA were specific, the labeled DSO dissociated from DSO-RepA complex when challenged with cold DSO. Thus, it is likely that generation of OC of pTS236-K was due to introduction of a nick in the predicted DSO (Fig. 4b). In plasmids replicating through RC mode, a considerable amount of single-stranded (SS) intermediate is always seen in actively grown cultures^36^. Before designating pTS236 as RC plasmid with reasonable confidence, experiments were conducted to detect the SS form of pTS236 in cultures of *Acinetobacter sp*. DS002 following established procedures^37^. As seen in the RC plasmids, a strong signal was obtained only in the plasmid samples that were not treated with S1 nuclease. Since S1 nuclease treatment preferentially eliminated SS form of pTS236 no such signal was seen, giving a clear indication that pTS236 is a RC plasmid (Fig. 5).

### The pTS236 is a homologue of Sphinx 2.36

Although database searches to find homologues of pTS236 identified *repA* genes from similar RC plasmids such as pPP81^31^, a clear overall sequence similarity was only seen with the recently reported Sphinx 2.36 DNA sequences. About 67% identity was found between these two circular DNA molecules. The DSO sequences of both plasmids were identical and they were found to be highly similar to the DSOs of other RC plasmids (Fig. 4a). Interestingly, the Sphinx 2.36 plasmid also codes for Orf106 and Orf96 homologues (Supplementary Fig. S4). Though existence of *orf96* was not predicted in the reported sequence, a thorough analysis of the Sphinx 2.36 sequence revealed its presence immediately downstream of the *orf106* gene. As seen in pTS236, the two ORFs in Sphinx 2.36 appear to be translationally coupled, suggesting functional conservation of the gene products.

Despite sharing identical DSO sequences, the RepA sequences were not so highly conserved between pTS236 and Sphinx 2.36 (Supplementary Fig. S1, S4). Only 61% similarity was seen between these two RepA amino acid sequences encoded by these two circular DNA molecules. This observation prompted us to reanalyze the *repA* sequence of Sphinx 2.36 to identify possible frame-shifts in the sequence. Initially, the proteins coded in all six frames of the *repA* sequence were aligned with the RepA sequence of pTS236. The protein encoded by frame + 2 was 47% similar to the N-terminal part of pTS236 RepA. Similarly, the protein encoded by frame + 1 was again similar (43%) but with the C-terminal portion of pTS236 RepA. The RepA sequence reported by Manuelidis was encoded by frame + 3 and it matched the RepA of pTS236 from amino acids 48 to 281^26^. Reconstruction of the RepA-coding sequences into a single frame by arbitrarily changing the sequence revealed a gene product with 57% identity to the RepA of pTS236. Interestingly, the Sphinx 2.36 *repA* also has a predicted UUG translation initiation codon, along with a putative promoter overlapping DSO (Supplementary Fig. S4). Taken together, these data suggest possible frame-shifts in the coding region, which could result either from sequencing errors or mis-incorporation of bases while amplifying the Sphinx 2.36 sequence by using ø29-polymerase from infected brain samples^26^. Although no data is available to prove that the predicted RepA of Sphinx 2.36 is functional, nevertheless, if the experimental evidence shown in this study is taken into consideration, it suggests that RepA initiated from the alternative UUG codon might be needed for replication of Sphinx 2.36 in neuronal cells. A better understanding will only be possible through a new analysis of the sequence of Sphinx 2.36 *repA*.

### pTS236 is the genome of a phage

**Figure 6:**
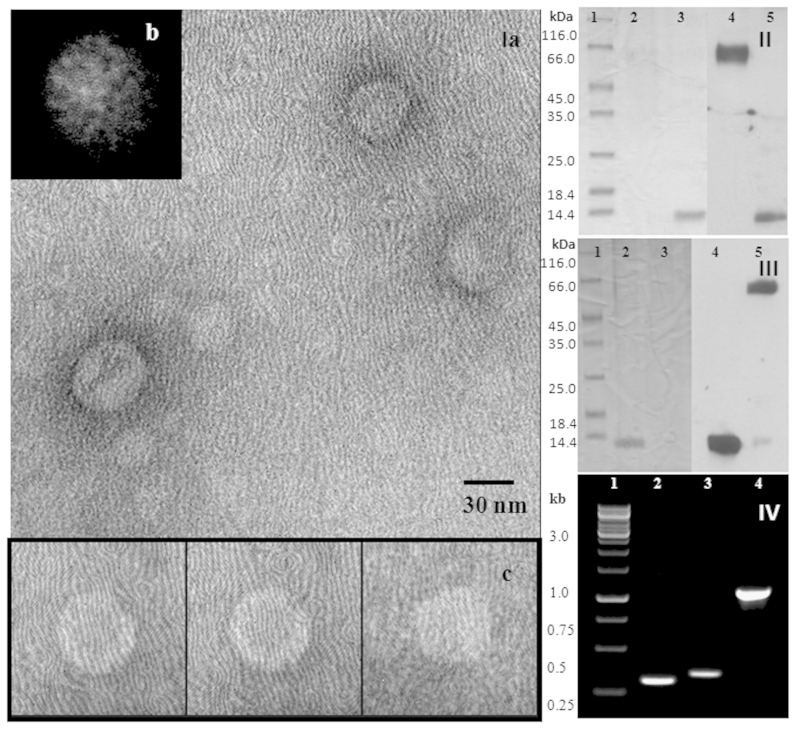
Panel I (a): TEM image of phage AbDs1. Out of the 50 isolated particles used for generating a class average, a few selected virions are shown in panel (c). The icosahedral features that are seen in the class average generated using the EMAN software tool, confirms the formation of virions (panel b). Panel (II) shows a western blot analysis of immune-purified phage AbDs1 using Orf96-specific antibodies. Lane 1 represents the molecular mass marker, lanes 2 and 3 are loaded with phage proteins and recombinant Orf96, respectively. Lanes 4 and 5 show the corresponding western blots. The western blot performed using Orf106-specific antibodies is shown in panel (III). Lanes 2 and 3 represent recombinant Orf106 and proteins of phage AbDs1, respectively. Lanes 4 and 5 show the corresponding western blots. Amplification of pTS236-specific ORFs from immune-purified AbDs1 phage particles is shown in panel (IV). Lane 1 represents kilo base ladder. Lanes 2, 3 and 4 show amplification of *orf96*, *orf106* and *repA*, respectively.

Due to existence of a pTS236 homologue in brain samples of TSE-infected animals we have done further experiments to determine whether pTS236 is a genome of a phage infecting *Acinetobacter* sp. DS002 cells. The PEG precipitate obtained from the culture supernatant was taken and immuno-purified (IP) by passing through a protein-A column conjugated with polyclonal antibodies raised against Orf96. The pure affinity-purified particles were then examined by transmission electron microscopy (TEM). Figure 6 shows the electron micrograph of icosahedral virions (Panel - Ia). From among these virions, a total of about 50 isolated particles (some selected virions are shown in Fig. 6, panel Ic) were used for generating a class average using the EMAN software tool^38^ and this is shown in Fig. 6, panel - Ib. The icosahedral features that are seen in the class average (Fig. 6, panel Ib) confirms the formation of virions. In support of this data the immuno-purified phage particles also gave a signal when western blots were performed using Orf96 and Orf106 specific antibodies (Fig. 6, panels II, III). The obtained signals were found to be bigger than the recombinant Orf96 and Orf106, indicating formation of SDS-resistant Orf96 and Orf106 multimers (Fig. 6, panels II, III). Multimerization of Orf96 and Orf106 was also evident when western blots were performed for cellular proteins extracted from *Acinetobacter* sp. DS002 cells. A Orf96-specific ladder-like signals were seen in westerns blots (Supplementary Fig. S5). Even the PCR reaction performed using immuno-purified phage particles as template gave pTS236-specific amplicons suggesting the existence of pTS236 in the icosahedral-shaped phage particles (Fig. 6, panel IV). Identical results were obtained when a PIP (phage immuno-purification) assay was repeated using Orf106 antibodies. However, neither phage particles nor pTS236-specific amplifications were seen when similar assays were performed using an unspecific anti-organophosphate hydrolase (OPH) antibody. All these results confirm the specificity of the PIP (phage immuno-purification) assays and clearly indicate that the Orf96 and Orf106 are the coat proteins of isolated phage.

**Figure 7:**
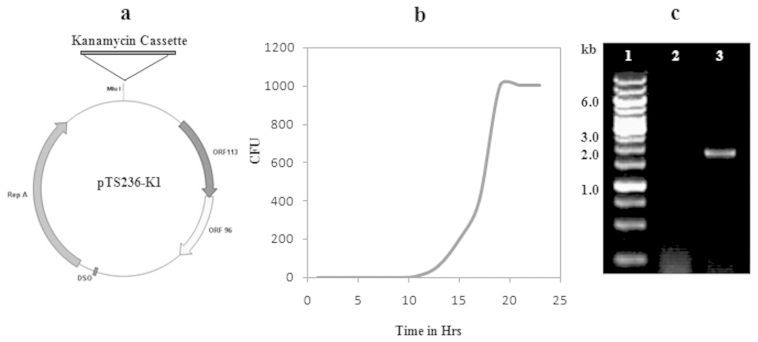
Panel (a) indicates physical map of pTS236-K1. Panel (b) indicates the generation of kanamycin resistant *Acinetobacter* sp. DS002 colonies after infection with AbDs1 (pTS236-K1). Amplification of the kanamycin gene from immune-purified phage particles, AbDs1 (pTS236-K1), is shown in panel (c). Lane 1 represents the kilobase ladder, lanes 2 and 3 are loaded with the PCR-amplified kanamycin gene from AbDs1 and AbDs1 (pTS236-K1).

While gaining further information on the infectivity of identified phage, the *Acinetobacter* sp. DS002 was transformed with pTS236 variant pTS236-K1. The immuno-purified phage particles from the culture supernatant of *Acinetobacter* sp. DS002 (pTS236-K1) cells were re-infected into wild type cells of *Acinetobacter* sp. DS002. The wild type *Acinetobacter* sp. DS002 gave kanamycin-resistant colonies after 11 hours of infection (Fig. 7b). No such colonies were observed in *Acinetobacter* sp. DS002 cells incubated with purified pTS236-K1 DNA even at a concentration of 1 μg/ml in LB medium.

## Discussion

We initiated the current study to gain clearer insights into the plasmid profile of *Acinetobacter* sp. DS002 isolated from agricultural soils polluted with OP insecticides. The circular DNA, pTS236 was the smallest of the rescued plasmids of *Acinetobacter* sp. DS002. Analysis of its sequence led to the discovery of a link between pTS236 and Sphinx 2.36. The Sphinx 2.36 sequence matches throughout its length the sequence of pTS236 and shows an overall 67% match at the nucleotide sequence level. In a recent network analysis, only the pTS236 homologue, p4ABAYE, showed no apparent link with existing *Acinetobacter* plasmids^13^. This lack of evolutionary linkage prompted the question as to whether pTS236 might be a replicative form of a phage genome propagating using *Acinetobacter* sp. DS002 as a host. We have therefore examined a polyethylene glycol precipitate of the spent medium by TEM. Our initial observations under TEM gave no indication of the presence of phage particles and hence it was assumed that pTS236 is a unique plasmid found in *Acinetobacter* sp. DS002 cells. However, further purification steps of the PEG precipitate of the spent medium enriched phage particles and the existence of icosahedral phage particles was confirmed (Fig. 6, panel I). These pure phage particles have also cross-reacted with Orf106 and Orf96 antibodies and the viral immune-precipitation (VIP) assay performed to amplify pTS236 from immune-purified viruses amplified pTS236-specific sequences (Fig. 6 II, III, IV). If all these results are taken into consideration, encapsulation of pTS236 DNA is evident in an icosahedral protein coat generated by Orf106 and Orf96.

*Acinetobacter* sp. are robust microbes that can adapt to a variety of habitats, including soil. They can easily gain entry into animals while grazing or drinking water. In fact, they were implicated in TSE due to the existence of high levels of *Acinetobacter*-specific antibodies in infected animals that tested BSE-positive^39^. An amino acid sequence similarity has also been identified between a bovine prion sequence (RPVDQ) and uridine diphosphate-N-acetyl glucosamine-1-carboxy-vinyl transferase of *A. calcoaceticus*. In further support of such an unusual observation, even the class specific antibodies were significantly elevated against structurally related synthetic peptides^40^. Following these important observations, serious efforts were made to find a possible epidemiological link between BSE and *Acinetobacters*. However, no solid evidence was found to support this decade-old proposition.

Prion involvement in BSE has been proved beyond doubt^18^,^19^, and in all TSE-infected animals, the prion protein has an amyloid fold resistant to proteases. A major unsolved question in BSE is the identification of the factor or factors responsible for its misfolding. Though a ‘protein only’ theory has gained wider acceptance, detection of nucleic acids in TSE preparations supported the hypothesis that contradicts this otherwise established notion^25^,^41^. One school of thought which argues against the protein-only theory suggests involvement of a nucleic acid-based ‘cofactor’ during conversion of the cellular form of the prion, PrP^C^ into the disease-causing, protease-resistant PrP^Sc^ 42,43. In support of this notion, a vertebrate single-stranded RNA was shown to act as a ‘cofactor’ for the *in vitro* amplification of PrP^Sc^ 44. Though no viral involvement is shown in BSE, virus-like particles were found associating with BSE-infected samples^43^.

The present study was neither designed to gather evidence in support of a particular school of thought nor do the findings support any of the existing hypotheses. It rather provides strong evidence to link the Sphinx 2.36 sequence found in BSE-infected samples to the genome of *Acinetobacter* sp. DS002 phage. These results should be taken into account when considering the findings of Manuelidis who identified the existence of viral particles in BSE-infected samples with Sphinx 2.36 as its genome^26^. Moreover, BSE-infected samples should be thoroughly examined to determine whether samples have *Acinetobacter* contamination. Even if the possibility of *Acinetobacter* contamination can be ruled out, nevertheless the presence of *Acinetobacter* phage-specific circular DNAs in brain samples of infected animals is too convincing to be ignored. Though no direct involvement of Sphinx has been demonstrated in the generation of PrP^Sc^, their amplification, particularly in infected brain samples is potentially significant. Even in the absence of a causal link between Sphinx and TSE, the existence of a circular DNA in mammalian brain, which has a resemblance to a genome of an *Acinetobacter* phage is an important observation.

The existence of plasmid sequences homologous to the second Sphinx sequence, Sphinx 1.76, have been reported in *Acinetobacter baumannii* TCDC-AB0715^27^ and *Acinetobacter baumannii* AYE^13^. If the sequence similarity is seen in the light of the present findings, most likely the reported circular DNA as plasmids p1ABTCDC0715 and p2ABAYE can be considered to be genomes of phage particles.

## Methods

### Bacterial culture and gene manipulations

Bacterial strains and plasmids used in this study are listed in Table 1. The primer sequences are given in Supplementary Table S1. *Escherichia* *coli* cells were grown at 37°C in LB medium. *Acinetobacter* sp. DS002 was grown at 30°C either in LB medium or in minimal medium prepared by dissolving 4.8 g of K_2_HPO_4_, 1.2 g of KH_2_PO_4_, 1 g of NH_4_NO_3_, 0.2 g of MgSO_4_.7H_2_0, 0.04 g of Ca(NO_3_)_2_.4H_2_0 and 0.001 g of Fe_2_(SO_4_)_3_ in 1000 ml of distilled water. Succinate (10 mM) was supplemented as a carbon source. When necessary, antibiotics ampicillin (100 μg/ml), kanamycin (30 μg/ml), streptomycin (20 μg/ml) and chloramphenicol (30 μg/ml) were added to the growth medium. Indigenous plasmids were isolated from *Acinetobacter* sp. DS002 essentially by following the Currier-Nester protocol^45^. Molecular cloning, blotting techniques and electroporation of *E. coli* and *Acinetobacter* sp. DS002 were performed following procedures described elsewhere^46^. Detection of a single-stranded intermediate of pTS236 in *Acinetobacter* sp. DS002 was done by following procedures described elsewhere^37^. The 16 S rRNA gene cloning and phylogenetic tree construction was done as described previously^47^.

### Rescue-cloning of plasmids from *Acinetobacter* sp. DS002

Plasmids were rescue-cloned by tagging the mini-transposon with a R6Kγori replicative origin. The isolated plasmids were treated with plasmid safe (EPICENTRE Biotechnologies, USA) to remove linear DNA and were used to tag with EZ-Tn5™ <R6Kγori/KAN-2> using the Transposon Insertion Kit (EPICENTRE Biotechnologies, USA). A 10 μl reaction mixture contained 1 μl of 10 × buffer, 1 μg of plasmid DNA, an equimolar concentration of transposon and 1 U of transposase. The reaction mixture was incubated for 2 hours at 37°C and the reaction was stopped by adding 1 μl of stop solution followed by incubation of the reaction mixture at 70°C for 10 min. A 1 μl aliquot of the transposition mixture was then electroporated into *E. coli* EC100D *pir*-116 cells. The Kan^R^ colonies were independently subcultured in LB medium supplemented with arabinose (1 mM) and the rescued plasmids were isolated to obtain their restriction profile and those which gave an unique restriction profile were selected for sequence determination.

### Direct sequencing of rescue-cloned plasmid pTS236

After isolating mini-transposon-tagged *Acinetobacter* sp.DS002 plasmids, the rescue-cloned plasmids were sequenced using transposon-specific primers followed by primer walking. The sequence was analyzed using bioinformatic tools available online. The NCBI GLIMMER programme^48^ was used to predict open reading frames (ORFs) and for promoter prediction, BPROM software (www.softberry.com/berry.phtml?topic=bprom) was used. NCBI BLAST^49^, EBI ClustalW2^50^ and REPFIND software^51^ were used for sequence similarity search, multiple sequence alignment and for finding repeat sequences, respectively. The double-stranded origin (DSO) of replication was identified by aligning the pTS236 DNA sequence with known sequences of other rolling-circle plasmids available in the database^52^. DNA secondary structure was predicted using the Mfold programme^53^.

### Expression and purification of RepA

The strategy followed for expression of RepA from the predicted start codon UUG and from its first conventional AUG as shown in Fig. 2b. While expressing RepA from the predicted start codon UUG, the *repA* gene was amplified from plasmid pTS236 using the forward (DS00109) and reverse (DS00110) primers carrying EcoRI and XhoI restriction recognition sequences, respectively. The *repA* amplicon was then digested with EcoRI and XhoI and ligated into pET23b digested with the same enzymes. The cloning strategy places *repA* in-frame with the vector-encoded His-tag of pET23b and the recombinant plasmid pTRW1, which encodes RepA with a C-terminal His-tag. The RepA_6HIS_ induced in *E. coli* BL21 (pTRW1) was affinity-purified by using a Ni-Sepharose High Performance column (XK16/20, GE Healthcare) following procedures described elsewhere^54^. A similar strategy was followed while cloning of *repA* from its first AUG except that the primers DS00111 and DS00110 were used as forward and reverse primers and the resulting expression plasmid is named as pTRW2. In both the cases start and stop codons were modified to facilitate incorporation of EcoRI and XhoI sites at 5′ and 3′ ends of *repA* sequence.

### Site-directed mutagenesis

Site-directed mutagenesis was performed following the protocol described elsewhere^55^. To replace the codon specifying tyrosine in *repA* with the codon specifying phenylalanine, oligos DS00103 and DS00104 were used. Similarly, oligos DS00105/DS00106 and DS00107/DS00108 were used to introduce termination codons in all frames immediately downstream of the start codon AUG of *orf106* and *orf96*, respectively. In all PCR reactions plasmid pTS236-K was used as template. After performing standard PCR using *Pfu*-polymerase the amplicons were digested with DpnI and the DpnI-resistant plasmids were transformed into *E. coli* *pir*116 cells and the presence of the desired mutation was confirmed by determining the sequence of the entire gene.

### Determination of RepA/DSO interactions

Both electrophoretic mobility shift assay (EMSA) and RepA-dependent conversion of supercoiled (SC) pTS236 to the open circular (OC) form were used to determine RepA-DSO interactions. The gel extracted supercoiled pTS236-K was used as substrate to determine RepA-dependent nick generation. About 0.8 μg of supercoiled pTS236 was incubated at 32°C with 170 ng of RepA in a reaction mixture (30 μl) containing 10 mM Tris-HCl (pH 8.0), 100 mM KCl, 10 mM Mg(OAc)_2_, 1 mM EDTA, and 10% (w/v) ethylene glycol. The reactions were stopped by adding 4 μl of 20% (w/v) EDTA at different time intervals and samples were analyzed on 0.8% (w/v) agarose gels. A similar analysis was made by incubating the supercoiled pTS236-K with increasing concentrations of RepA. The RepA-dependent conversion of pTS236-K (SC) to pTS236-K (OC) was identified based on changes observed in the band intensities of the SC and OC forms of pTS236-K in dependence on time and protein concentration.

EMSA was performed as described previously^56^. A 100 bp DNA fragment containing the DSO region was amplified using the primer pair DS00101 and DS00102 and the amplicon was end-labeled following the standard protocols^46^. The following amounts (0 ng, 100 ng, 250 ng, 500 ng and 1000 ng) of purified RepA_6His_ were incubated with 2 picomoles of the labeled probe in 20 μl of binding buffer (20 mM Tris-HCl [pH 8.0], 1.0 mM EDTA, 6 mM MgCl_2_, 50 mM KCl, 50 μg/ml bovine serum albumin [BSA], and 5% (w/v) glycerol) containing 5 μg/ml of herring sperm DNA for 20 min at 25°C. The DNA-protein mixture was resolved on a 5% (w/v) native polyacrylamide gel and was analyzed by autoradiography.

### Phage immuno-purification (PIP)

Polyclonal antibodies against recombinant Orf96_6His_ and Orf106_6His_ were raised in male New Zealand (O.B) rabbits following standard procedures. The polyclonal antibodies raised against these two recombinant proteins were affinity purified and used for performing western blots and immuno-purification (IP) experiments. After establishing the specific reactivity of the antibodies with their respective antigens they were independently conjugated to a protein-A column. The culture supernatant collected from *Acinetobacter* sp. DS002 cultures grown for 24 hrs were PEG-precipitated by bringing the culture supernatant to a final concentration of 10% PEG and 1 M sodium chloride. After leaving the mixture overnight at 4°C it was centrifuged at 15, 000 rpm for 20 min to collect the precipitate. The precipitate was then re-dissolved in 20 mM sodium phosphate buffer, pH 7 and passed through protein-A columns linked to either polyclonal antibodies of Orf96_6His_ or Orf106_6His_. The columns were washed thoroughly with 20 mM sodium phosphate buffer, pH 7 and the particles bound to columns were eluted by passing Glycine- HCl, pH 2.7. The bound material collected was then used to perform western blots using both Orf96 and Orf106 antibodies and to amplify ORFs encoded by particles bound to the affinity column.

### TEM images of phage AbDs1

The sucrose gradient-purified virions were adsorbed to a freshly glow-discharged 200 mesh carbon coated copper grid (Ted Pella Inc. USA) and stained with an aqueous solution of uranyl acetate (2% w/v). Electron micrographs of these virions were recorded on a FEI Technai G^2^ SuperTwin 200 instrument operating at an acceleration voltage of 120 kV. A magnification of 19,500 × with an effective magnification of 100 nm at the specimen level was used to record the micrographs. The images were recorded using a Gatan Orius 803 digital camera (2 k × 2 k) corresponding to 3.422 Å/pix at the specimen level as calibrated with the help of the calibration grid (Electron Microscopy Sciences, Hatfield, PA 19440, USA). A total of about 50 isolated particles (some selected virions are shown in Fig. 6, panel Ic) were taken for generating a class average using the EMAN software tool^38^ and to see the icosahedral features, typically seen during the formation of virions.

### Infection assay

The pTS236 has unique Mlu I site in the intergenic region of *repA* and *orf106*. The kanamycin cassette amplified from pUC4K^57^ was amplified using primers, DS00112 and DS00113, appended with a Mlu I recognition site. This amplicon was then cloned into the Sphinx 2.36 homologue, pTS236 digested with the same enzyme. The recombinant circular DNA, named as pTS236-K1 (Fig. 7a) was then electroporated into *Acinetobacter* sp. DS002 cells and selected on kanamycin-containing agar plates. The supernatant collected from the kanamycin-resistant cultures of *Acinetobacter* sp. DS002 (pTS236-K1) was PEG-precipitated and passed through a protein-A column conjugated with Orf96_6His_ specific polyclonal antibodies. After repeated washings, the bound material was eluted as described in earlier sections and used to seed mid-log phase *Acinetobacter* sp. DS002 cells. A portion of culture was withdrawn every hour and serially diluted culture was then plated on kanamycin-containing agar plates to observe the generation of kanamycin-resistant colonies. A graph was plotted overtime on the X-axis against the number of kanamycin-resistant CFUs on the Y-axis (Fig. 7b). The affinity-purified phage particles from *Acinetobacter* sp. DS002 (pTS236-K1) were used as template to amplify the kanamycin-resistance gene (Fig. 7c).

## Author Contributions

D.S. conceived and designed the study. T.L., S.K., G.P. executed the work. V.R.M., S.A., G.K. and M.A. purified the virions and obtained TEM. images. D.S., T.L., S.K. and G.K. wrote the manuscript.

## Supplementary Material

Supplementary Information Acinetobacter phage genome is similar to Sphinx236, the circular DNA copurified with TSE infected particles

## Figures and Tables

**Table 1:** Bacterial strains and plasmids

S.N.	Strain/Plasmid/Primer Name	Genotype/Phenotype/Sequence Description	Reference/Source/Remark
	***Strains***		
1	*E. coli* DH5α	*fhuA2* Δ*(argF-lacZ)U169 phoA glnV44 *Φ*80* Δ*(lacZ)M15 gyrA96 recA1 relA1 endA1 thi-1 hsdR17*	58
2	*E coli* BL21 DE3	*hsdS gal(λcIts857 ind1 sam7 nin5lac UV5 T7 gene 1*	59
3	*E. coli* pir116	*F^-^ mcrA Δ(mrr-hsdRMS-mcrBC) φ80dlacZΔM15 ΔlacX74 recA1 endA1 araD139 Δ(ara, leu)7697 galU galK λ- rpsL (Str^R^) nupG pir-116(DHFRv)*	EPICENTRE Biotechnologies
4	*Acinetobacter sp.* DS002	Sm^r^ and Cm^r^	This work
	***Plasmids***		
5	pET23b	Ap^r^, Expression vector	Novogen
6	pTRW1	Ap^r^, *repA* cloned in pET23b from predicted start codon TTG	This work
7	pTRW2	Ap^r^, *repA* cloned in pET23b from its first ATG codon	This work
8	pTRM	Expression plasmid coding for RepA Y265F	This work
9	pTS236K	Rescued cloned pTS236 having minitransposon in the intergenic region of *repA* and *orf96*	This work
10	pTS236K1	pTS236 having kanamycin cassette at unique Mlu I site found in the intergenic region of *repA* and *orf106*	This work
11	pT106M	pTS236-K having termination codon immediately downstream to the start codon of *orf106*	This work
12	pT96M	pTS236-K having termination codon immediately downstream to the start codon of *orf96*	This work
