# Supplementary material for: Acinetobacter phage genome is similar to Sphinx 2.36, the circular DNA copurified with TSE infected particles
Source: Sci Rep. 2013 Jul 19;3:2240. doi: 10.1038/srep02240 (PMC3715749; doi:10.1038/srep02240)
Supplement: Supplementary Information — Acinetobacter phage genome is similar to Sphinx236, the circular DNA copurified with TSE infected particles [file srep02240-s1.doc]

***Acinetobacter* phage genome is similar to Sphinx236, the circular DNA copurified with TSE infected particles**

Toshisangba Longkumer, Swetha Kamireddy, Venkateswar Reddy Muthyala, Shaikh Akbarpasha, Gopi Krishna Pitchika, Gopinath Kodetham^1^ Murali Ayaluru^2^

and

Dayananda Siddavattam*

Department of Animal Sciences, ^1^Dept. of Plant Sciences, School of Life Sciences, University of Hyderabad, Hyderabad – 500 046, India.

^2^Bioinformatics Centre, Pondicherry University, Puducherry - 605 014, India

*Address for correspondence:

Dr. Dayananda Siddavattam

Dept. of Animal Sciences, School of Life Sciences,

University of Hyderabad, Hyderabad – 500 046.

Phone: ++91-40-23134578

Fax : ++91-40-23010120

Email: sdsl@uohyd.ernet.in


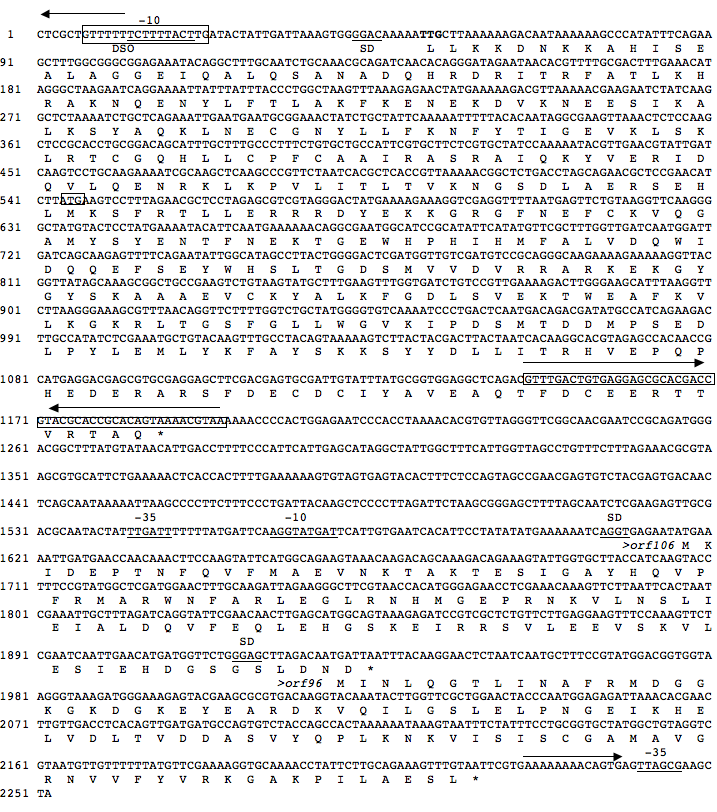


**Supplementary Figure S1.** Nucleotide and deduced amino acid sequence of plasmid pTS236. The putative DSO is shown as an open box. The consensus σ^70^ promoters and RBS sequences are underlined. The start codon UUG is indicated in bold. The codon AUG specifying methionine 165 is indicated as an open box. The putative SSO is indicated as an open box.


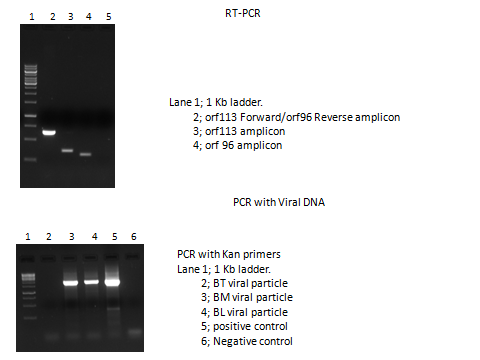


**Supplementary Figure S2.** Transcriptional organization of *orf106* and *orf96*. The RT-PCR was performed using *orf106*-specific forward and *orf96*-specific reverse primers and is shown in lane 2. Lanes 3 and 4 represent RT-PCR done using ORF-specific primers. Lane 5 represents the negative control.


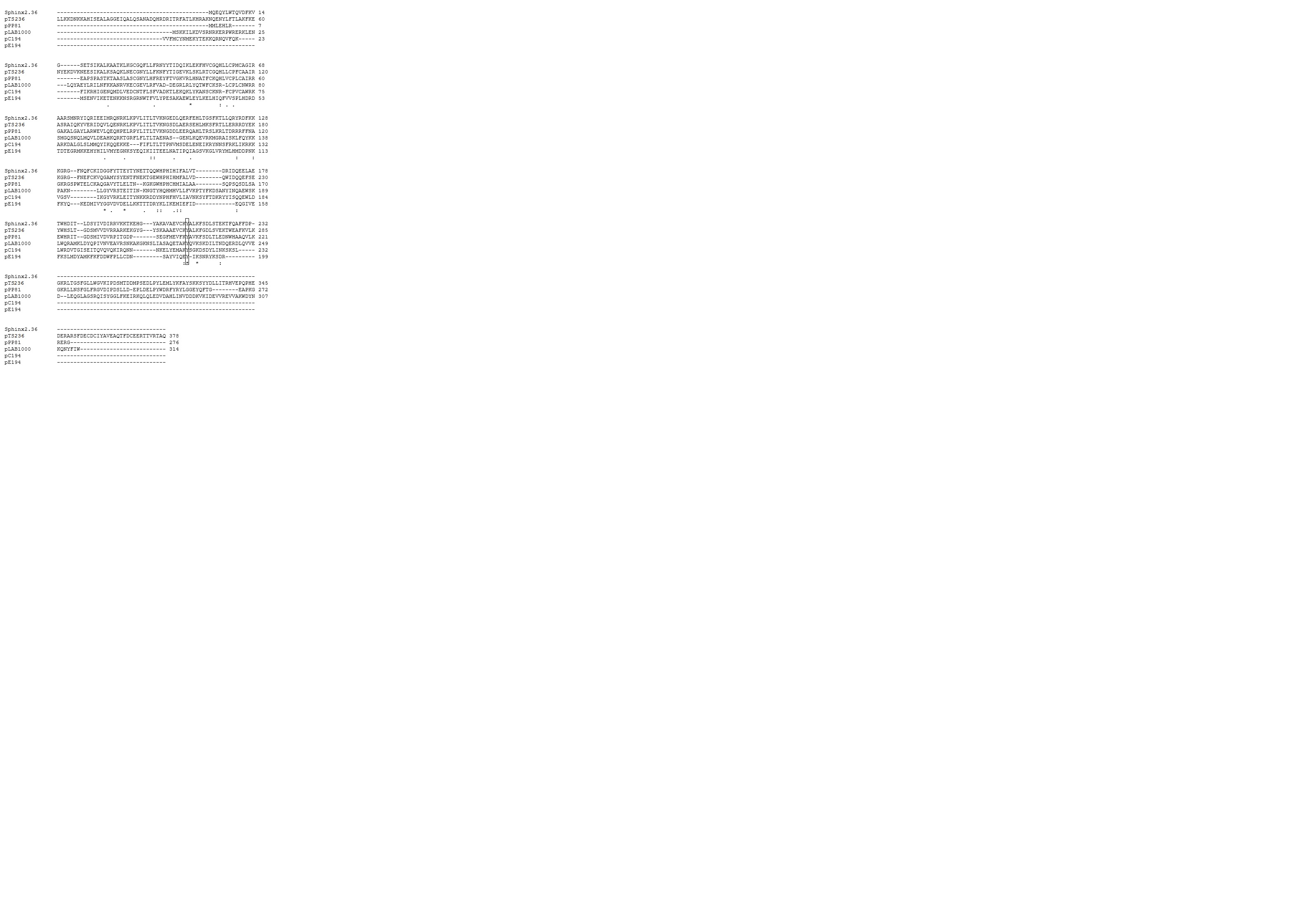


**Supplementary Figure S3.** Multiple sequence alignment of RepA sequences of pTS236 and Sphinx 2.36 with RepA sequences of other rolling-circle replicating plasmids, pPP81 (CAC01244.1), pC194 (NP_040435.3), pLAB1000 (AAA98163.1), pT181 (AAA26033.1) and pE194 (AAA25603.1) isolated from *Pseudomonas* *putida*^31^, *Staphylococcus* *aureus*^33^, *Lactobacillus* *hilgardii* ^35^, *S. aureus* and *S. aureus* ^34^, respectively*.* The conserved tyrosine residue involved in initiation of replication is shown as an open box.


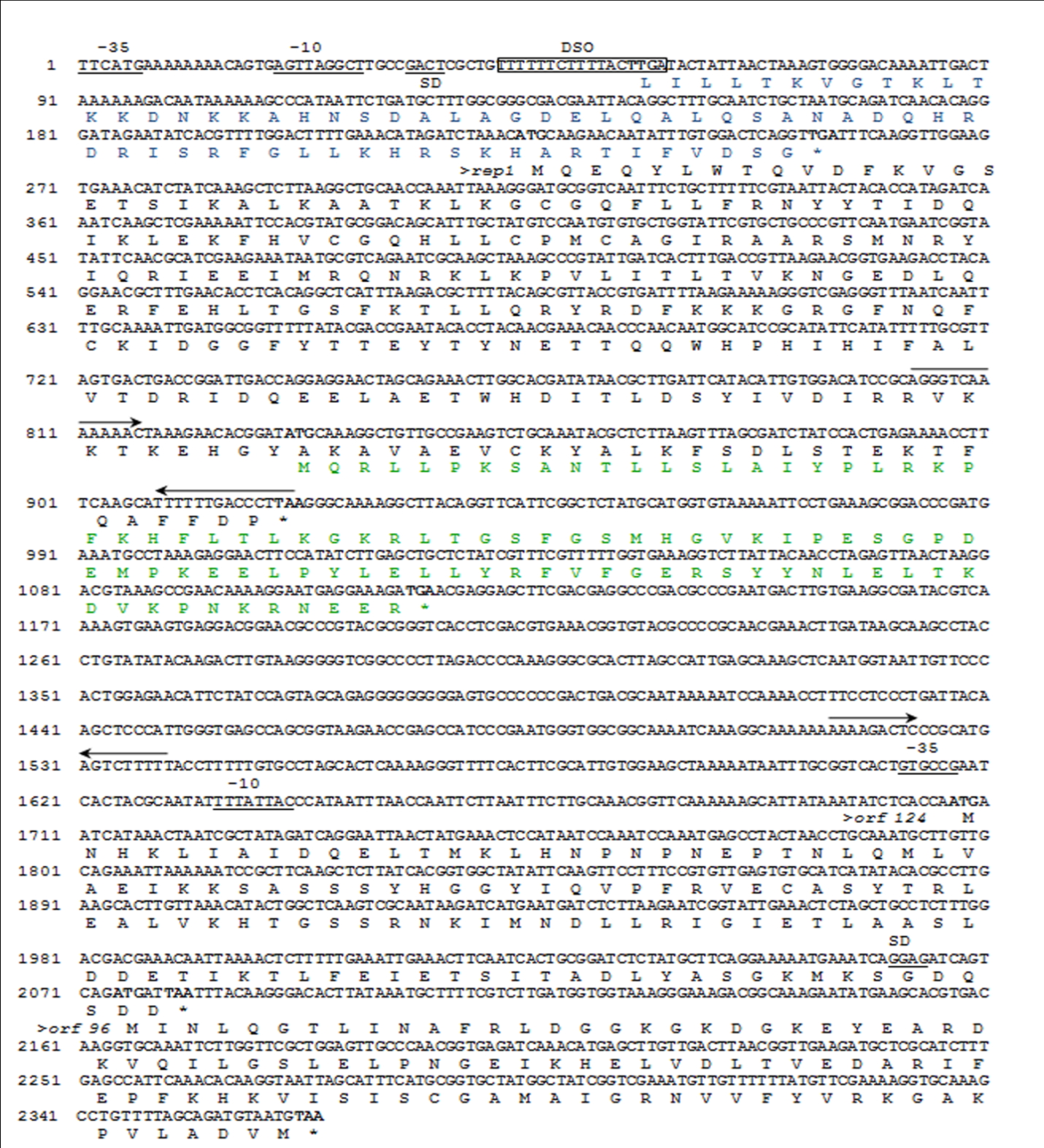


**Supplementary Figure S4.** Nucleotide and deduced amino acid sequence of Sphinx 2.36. The protein sequences coded by different frames of *repA* are shown. The protein showing homology to the N-terminus of RepA of pTS236 is represented in blue. The central portion of RepA found to be similar both in Sphinx 2. 36 and RepA of pTS236 is shown in black letters. The protein predicted from the +1 frame and which shows homology to the C-terminous of RepA of pTS236 is indicated in green. DSO and transcriptional terminators are shown with an open box and inverted arrows, respectively. Putative promoter and SD sequences are underlined.

**Supplementary Table S1.**

| **S.No** | **Primer Name** | **Sequence [Forward Primer(F*)/ Reverse Primer (R*)]** | **Remark** |
| --- | --- | --- | --- |
| 1  2  3  4  5  6  7  8  9  10  11  12  13  14 | DS00101  DS00102  DS00103  DS00104  DS00105  DS00106  DS00107  DS00108  DS00111  DS00110  DS00109  DS00110  DS00112  DS00113 | GCAGAAAGTTTGTAATTCG (F*)  GCTTCTGAAATATGGGCTT (R*)  GTCTGTAAGTTTGCTTTGAAG (F*)  CTTCAAAGCAAACTTACAGAC (R*)  CATTCCTATATATGTAATGATCAGGTG (F*)  CACCTGATCATTACATATATAGGAATG (R*)  GAGCTTAGACAATGTGATAATTACAAGG(F*)  CCTTGTAATTATCACATTGTCTAAGCTC (F*)  CCGAGAATTCTATGAAGTCCTTTAG (F*)  CCCTCGAGCTGTGCGGTGCGTACGG (R*)  GTGGGGGAATTCGATGCTTAAAAAAGAC(F*)  CCCTCGAGCTGTGCGGTGCGTACGG (R*)  GATCACGCGTTGTAGGTGGACCAGTTGGTGATT(F*)  GATCACGCGTTCACACAGGAAACAGCTATG(R*) | Primers used for amplification of DSO  Primers used to generate RepA Y265F  Primers used to introduce stop codon in *orf106*  coding region  Primers used to introduce stop codon in *orf96*  coding region  Primers used to amplify *repA* from its ATG  Primers used to amplify *repA* from TTG  Primers used  to amplify kanamycin gene from pUC4K as Mlu I fragment |


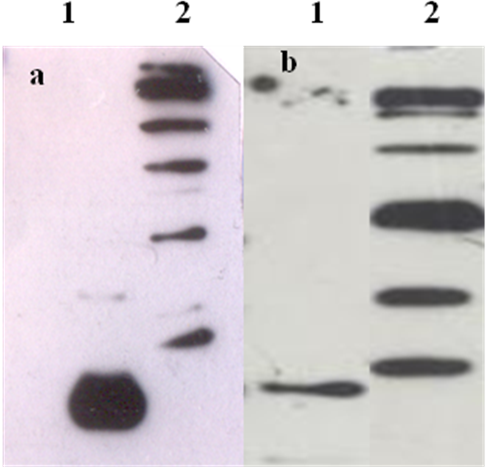


**Supplementary Figure S5.**

Panel a and b indicate western blots performed using Orf96- and Orf106-specific antibodies, respectively. Crude extracts prepared from cell lysates (lane 2), recombinant Orf96 (panel a, lane 1) and recombinant Orf106 (Panel-b, lane 1) were probed either with Orf96- (panel a) or with Orf106-specific antibodies (panel b). Ladder-like signals indicating multimerization of Orf96 and Orf106 were seen in protein extracts prepared from *Acinetobacter* sp. DS002 cells.
